# Predictors of Poor Postoperative Outcomes in Pediatric Surgery Patients in Rural Ghana

**DOI:** 10.1186/s12893-020-00867-9

**Published:** 2020-09-22

**Authors:** Sarah Peiffer, Anna E. Ssentongo, Laura Keeney, Forster Amponsah-Manu, Richard Yeboako, Richard Ofosu-Akromah, Temitope Ebenezer Arkorful, Eric Agyemang, Anthony Tsai, John Oh, Paddy Ssentongo

**Affiliations:** 1 Penn State University College of Medicine, 500 University Drive, Hershey, PA 17033 USA; 2 Eastern Regional Hospital, P.O. Box 201, Koforidua, Ghana

**Keywords:** Pediatric surgery, Outcomes, Morbidity, Mortality, Africa, Global health

## Abstract

**Background/Purpose:**

Perioperative complications cause significant pediatric morbidity and mortality in low- and lower middle -income countries. This study investigates factors associated with prolonged length of stay, 90-day readmission and in-hospital mortality among pediatric patients at Eastern Regional Hospital (ERH) in Ghana.

**Methods:**

This is a retrospective review of perioperative morbidity and mortality in children < 18 years at ERH in Koforidua, Ghana. All pediatric surgeries performed between January 2015 and December 2017 were included in this study. Univariate analysis was performed using Pearson’s chi-square tests or Fisher’s exact tests. Variables that were significant on univariate analysis were included in multivariable logistic regression models adjusted for age and gender.

**Results:**

We analyzed 468 patients < 18 years of age with a median length of stay (LOS) of 3 days. The 90-day readmission and in-hospital mortality rates were 138 and 17 per 1000 patients, respectively. The most common procedures were herniorrhaphy (19 %) and appendectomy (15 %). Gastrointestinal surgery, surgical trauma, surgical infection and lack of insurance were significantly associated with prolonged LOS. Young age and female gender were significantly associated with in-hospital mortality. Malaria was significantly associated with 90-day readmission.

**Conclusions:**

Malaria infection is a significant risk factor for readmission, which should be investigated and treated in pediatric surgical patients in rural Ghana. Ensuring that all patients have insurance may result in shorter hospital stays. Provision of laparoscopic equipment may reduce hospital stays for patients undergoing gastrointestinal surgery. Expansion of the surgical work force, particularly pediatric surgeons, could improve perioperative survival in the very young population.

**Level of Evidence:**

Retrospective comparative study.

## Background

Despite overall gains in global health, the burden of surgical diseases and complications in low- and lower middle- income countries (LMICs) remains high. In 2018, one in five surgical patients in LMICs had poor postoperative outcomes, such as surgical site infection (SSI), wound complication, or death [1]. An estimated 5 billion people do not have timely access to safe, affordable surgery [2]. The most substantial unmet need for surgical care is found in sub-Saharan Africa [2], in-which 43 % of the population is under 15 years old [3]. The burden of surgical disease is high within the pediatric population [4], accounting for 6–12 % of pediatric admissions in sub-Saharan Africa [5] with an overall mortality rate of 12 % [6]. Unmet pediatric surgical need is a significant contributor to death and disability [5].

Pediatric surgery presents unique challenges which make it distinct from adult general surgery. Children present with different surgical pathologies, respond differently to anesthesia, and have special perioperative needs associated with high perioperative mortality [5, 7]. The vast majority of research on perioperative morbidity and mortality is from developed countries [7–12], and the number of studies that have looked at the influence of medical comorbidities unique to sub-Saharan Africa are limited [13–16]. Pediatric surgery in sub-Saharan Africa is disadvantaged by limited resources, high numbers of sick patients relative to the number of providers, and high frequency of delayed presentations with advanced pathologies [13]. Lack of finances and transportation are significant barriers to care, and cultural preference for traditional medicines and home remedies may significantly delay patient presentations [4, 17].

Previous studies have found that risk factors for perioperative mortality in sub-Saharan Africa include neonatal age group, delayed presentation, emergency surgery, high American Society of Anesthesiologists (ASA) status, and multiple operative procedures [6, 18, 19]. Other studies have suggested that increased postoperative complication rates occur with poor nutrition [20] and medical comorbidities such as HIV infection [15]. However, factors associated with pediatric surgical mortality and poor postoperative outcomes in sub-Saharan Africa may vary greatly across the continent. Therefore, this study aims to investigate factors associated with prolonged length of stay, readmission within 90 days and in-hospital mortality among pediatric patients at the Eastern Regional Hospital in Ghana.

## Methods

### Study design

A retrospective review was performed on the medical records of all patients < 18 years old who were admitted to the surgical ward from January 2015 through December 2017 at the Eastern Regional Hospital (ERH) in Koforidua, Ghana. ERH is a referral center for 26 district hospitals in the Eastern Region and has a surgical volume of over 2500 cases per year. Like many other regional and district hospitals in Ghana, ERH has no pediatric surgical specialists so all pediatric cases are operated on by general surgeons. ERH serves a catchment area with a population of approximately 3 million, more than 40 % of which are children. A secondary analysis was conducted to examine possible associations between gender, insurance status, surgical procedure, malaria, anemia, white blood cell count (WBC), congenital or acquired conditions, type of procedure, length of stay, readmission and mortality. The study protocol was reviewed and approved by the internal review board of Penn State Milton S. Hershey Medical Center and by the ethical review board at ERH. In addition, this study was evaluated and approved by ERH for cultural appropriateness.

### Study population

The study subjects were identified through billing records using patient identification numbers recorded in the ERH administration and management system. All patients < 18 years old who had surgery at ERH from January 1, 2015 to December 31, 2017 were included in this study. Patients were excluded from the analysis if they were admitted to the surgical ward but did not undergo a surgical procedure. The authors acknowledge that in Ghana only children < 12 years old are considered pediatric patients, however the authors elected to include patients up to age 17 in order to maximize the sample size and to allow for comparison to studies conducted in countries with broader definitions of pediatric patients.

### Review of patient records

From the electronic medical records, information for each study subject was obtained including age, gender, insurance status, date of admission, date of surgery, date of discharge, length of stay (LOS), diagnosis, type of procedure, date of death, preoperative hemoglobin, preoperative WBC count, preoperative serum glucose, HIV, malaria, date of readmission, reason for readmission, and surgical site infection. For each patient, the diagnosis was categorized as congenital or acquired. Surgical procedures were classified as gastrointestinal, genitourinary, injury, neoplasm, surgical infection (management of an infection, such as surgery for infection source control) or miscellaneous. Prolonged LOS in this study was defined as greater than the median LOS, which was 3 days, thus prolonged LOS was defined as an admission lasting greater than or equal to 4 days. For the purposes of data analysis, anemia was defined as Hgb < 12 mg/dL, leukpenia as WBC ≤ 4,500 cells per µL and leukocytosis as WBC ≥ 11,000 cells per µL. HIV and malaria tests were performed preoperatively during the initial surgical admission. Surgical site infections (SSI) included both superficial and deep incisional SSI as well as organ/space SSI identified either during the initial hospital admission, on outpatient follow-up visits or upon readmission.

### Statistical methods

A 2-tailed 2-sample Student's t-test or Wilcoxon rank-sum test was invoked to compare means or medians respectively for continuous outcomes variables between groups. We performed Pearson's chi-square tests or Fisher’s exact tests for categorical variables. Values are expressed as the mean ± standard deviation (SD) for normally distributed variables, as median and interquartile range for skewed distribution, and as counts and percentages for categorical variables. Values that were found to be significant on univariate analysis were included in multivariable logistic regression models using backward elimination method. All multivariable analyses were adjusted for age (which was centered by subtracting the mean age from raw age values) and gender. Point estimates are reported as odds ratios (OR) and 95 % confidence intervals (CI) for each outcome (prolonged LOS, in-hospital mortality and 90-day readmission). The predictive models were cross-validated using leave-one out-cross-validation methods [21]. To determine the discriminative power of the predictive models, the area under the receiver operating characteristic curve (AUC) was calculated [22]. Statistical analysis was performed using SAS, version 9.4; SAS Institute Inc and R software (R Core Team 2018). For all statistical tests, alpha level was set at 0.05.

## Results

During the study period, 595 pediatric patients presented to the surgical ward at ERH. A total of 127 patients were excluded due to lack of a surgical procedure. Analysis was performed on the remaining 468 patients, whose clinical attributes are summarized in Table 1 and Table 2. Their ages ranged from 3 days to 17 years with a mean age of 9.0 years (standard deviation (SD) ± 5.3 years). Ninety percent of the patients in this study were male (Tables 1, 2, 3). Of note, all of the patients tested for HIV in this study were found to be HIV negative. The median length of stay was 3 days (IQR ± 3 days). The 90-day readmission rate was 49 per 1000 patients. The in-hospital mortality rate was 17 per 1000 patients. The details of the children who died are provided in Table 4. Thirty-eight percent of the mortalities occurred in infants. Mortality in children older than 1 year was 9 per 1000 patients, which was much lower than in children less than or equal to 1 year, which was 235 per 1000 patients (p =  < 0.0001).

**Table 1:** Descriptive Statistics: Demographics

Attribute	Cohort (*n* = 468)
Age in years, mean (SD)	9.0 (5.3)
Age Range	
≤ 1 yr, n (%)	17 (3.6)
2–3 yrs, n (%)	79 (17)
4-8yrs, n (%)	138 (29)
9–13 yrs, n (%)	105 (22)
14–17 yrs, n (%)	129 (28)
Gender	
Male, n (%)	422 (90)
Female, n (%)	46 (10)

**Table 2:** Descriptive Statistics: Clinical Attributes

Attribute	Cohort (*n* = 468)
Malaria, n (%)	25/207 (12)
HIV, n (%)	0/130 (0)
Insurance, n (%)	356 (76)
Anemia, n (%)	127/194 (65)
Leukopenia, n (%)	13/194 (7)
Leukocytosis, n (%)	92/194 (47)
Congenital vs Acquired	
Acquired, n (%)	420 (90)
Congenital, n (%)	48 (10)
Procedure Category	
Gastrointestinal, n (%)	274 (59)
Genitourinary, n (%)	61 (13)
Injury, n (%)	71 (15)
Miscellaneous, n (%)	32 (7)
Neoplasm, n (%)	19 (4)
Surgical Infection, n (%)	11 (2)

**Table 3:** Descriptive Statistics: Outcomes

Outcome	Cohort (*n* = 468)
Length of stay, median (IQR)	3.0 (3.0)
In-hospital Mortality, n (%)	8 (2)
90-day Readmission, n (%)	23 (5)
Surgical Site Infection, n (%)	2 (0.4)

**Table 4:** Clinical Characteristics of Mortalities

Patient	Gender	Age	Insurance	Diagnosis	Time from Admission to Surgery (days)	Time to Death (Postop Day)
1	1	< 1 mo	Yes	Bowel obstruction due to congenital constricting bands	2	1
2	1	< 1 mo	No	Esophageal atresia	3	2
3	1	< 1 mo	Yes	Bowel ischemia	4	4
4	1	1–2 yrs	Yes	Intussusception	0	3
5	2	3–5 yrs	Yes	Intussusception	10	0
6	2	3–5 yrs	Yes	Intussusception	3	0
7	2	> 5 yrs	No	Hepatic abscess	8	32
8	2	> 5 yrs	Yes	Typhoid perforation	1	1

**Fig. 1:**
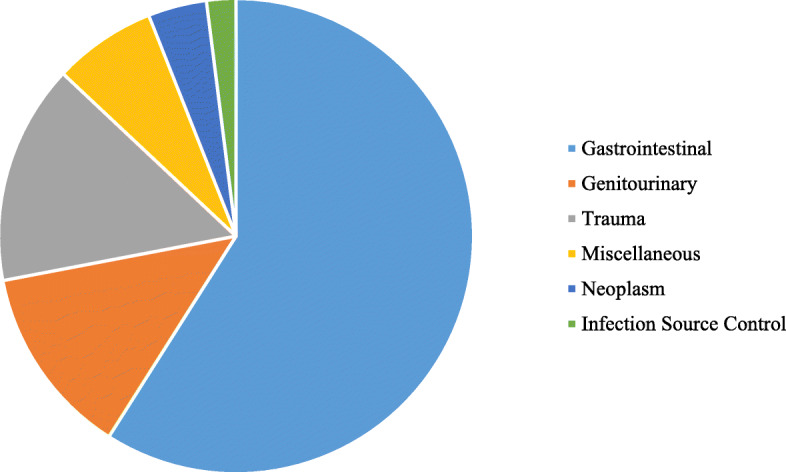
Distribution of Types of Pediatric Surgical Admissions

**Fig. 2:**
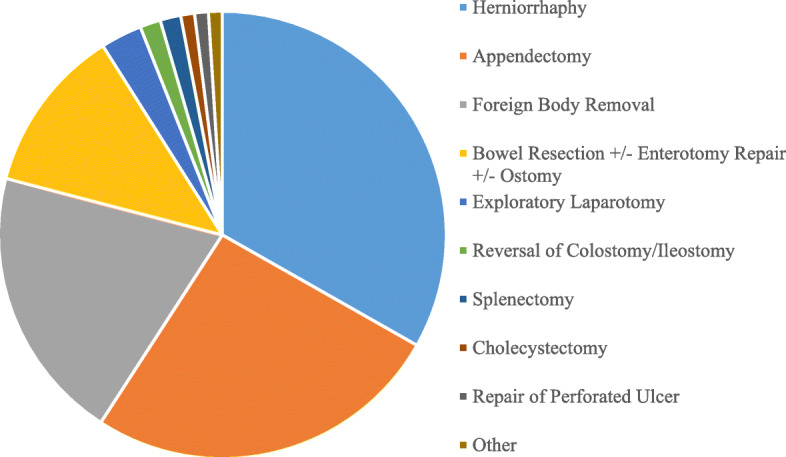
Distribution of Types of Gastrointestinal Procedures

The pediatric surgical conditions were categorized by whether they were congenital (10 %) or acquired (90 %). The distribution of the types of pediatric surgical conditions are shown in Fig. 1. Gastrointestinal surgery was the most common category, with the leading surgical procedures being herniorrhaphy (90/468, 19 %) and appendectomy (72/468, 15 %) (Fig. 2).

Summarized in Table 5 is the univariate analysis for the factors associated with prolonged LOS ≥ 4 days. Older age, lack of insurance, anemia (Hgb < 12 mg/dl), leukocytosis (≥ 11,000 leukocytes/µL), gastrointestinal surgeries and surgical management of infections were significantly associated with prolonged LOS. Similarly, summarized in Table 6 is the univariate analysis for predictors of in-hospital mortality. Younger age and female gender were significantly associated with in-hospital mortality. Similarly, summarized in Table 7 is the univariate analysis for predictors of 90-day readmission. Younger age and malaria infection were significantly associated with readmission within 90 days.

**Table 5:** Univariate Analysis for the Predictors of Prolonged Length of Stay Greater Than or Equal to 4 Days

Attribute	Cohort (*n* = 468)	Length of Stay < 4 days (*n *= 279)	Length of Stay ≥ 4 days (*n* = 189)	*p*Value
Age years, mean (SD)	468	8.5 (5.2)	9.6 (5.4)	**0.0256**
Gender				
Male (%)	422 (90)	253 (91)	169 (89)	0.6525
Female (%)	46 (10)	26 (9)	20 (11)	
Insured				
No (%)	112 (24)	52 (19)	60 (32)	**0.0011**
Yes (%)	356 (76)	227 (81)	129 (68)	
Malaria				
No (%)	182 (88)	105 (88)	77 (89)	0.8265
Yes (%)	25 (12)	15 (12)	10 (11)	
Anemia				
No (%)	67 (35)	40 (44)	27 (26)	**0.0095**
Yes (%)	127 (65)	51 (56)	76 (84)	
Leukopenia				
No (%)	181 (93)	83 (91)	98 (95)	0.2738
Yes (%)	13 (7)	8 (9)	5 (5)	
Leukocytosis				
No (%)	102 (53)	56 (62)	46 (45)	**0.0188**
Yes (%)	92 (47)	35 (38)	57 (55)	
Congenital vs Acquired				
Acquired (%)	420 (90)	246 (88)	174 (92)	0.1734
Congenital (%)	48 (10)	33 (12)	15 (8)	
Procedure Category				
Gastrointestinal (%)	274 (59)	151 (54)	123 (65)	**0.0011**
Genitourinary (%)	61 (13)	50 (18)	11 (6)	
Injury (%)	71 (15)	42 (15)	29 (15)	
Miscellaneous (%)	32 (7)	21 (8)	11 (6)	
Neoplasm (%)	19 (4)	12 (4)	7 (4)	
Surgical Infection (%)	11 (2)	3 (1)	8 (4)

**Table 6:** Univariate Analysis for the Predictors of Mortality

Attribute	Cohort (*n* = 468)	Alive (*n* = 460)	Died (*n* = 8)	*p*Value
Age years. mean (SD)	468	9.1 (5.2)	2.7 (3.0)	**0.0004**
Gender				
Male (%)	422 (90)	418 (91)	4 (50)	**0.0043**
Female (%)	46 (10)	42 (9)	4 (50)	
Insured				
No (%)	112 (24)	109 (24)	3 (38)	0.4042
Yes (%)	356 (76)	351 (76)	5 (62)	
Malaria				
No (%)	182 (88)	179 (88)	3 (100)	1.0000
Yes (%)	25 (12)	25 (12)	0 (0)	
Anemia				
No (%)	67 (35)	65 (34)	2 (40)	1.0000
Yes (%)	127 (65)	124 (66)	3 (60)	
Leukopenia				
No (%)	181 (93)	177 (94)	4 (80)	0.2957
Yes (%)	13 (7)	12 (6)	1 (20)	
Leukocytosis				
No (%)	102 (53)	100 (53)	2 (40)	0.6694
Yes (%)	92 (47)	89 (47)	3 (60)	
Congenital vs Acquired				
Acquired (%)	420 (90)	414 (90)	6 (75)	0.1936
Congenital (%)	48 (10)	46 (10)	2 (25)	
Procedure Category				
Gastrointestinal (%)	274 (59)	266 (58)	8 (100)	0.5798
Genitourinary (%)	61 (13)	61 (13)	0 (0)	
Injury (%)	71 (15)	71 (15)	0 (0)	
Miscellaneous (%)	32 (7)	32 (7)	0 (0)	
Neoplasm (%)	19 (4)	19 (4)	0 (0)	
Surgical Infection (%)	11 (2)	11 (2)	0 (0)

**Table 7:** Univariate Analysis for the Predictors of Readmission Within 90 Days

Attribute	Cohort (*n* = 468)	Not readmitted (*n* = 445)	Readmitted (*n* = 23)	*p*Value
Age years. mean (SD)	468	9.1 (5.3)	7.0 (4.7)	**0. 0494**
Gender				
Male (%)	422 (90)	399 (90)	23 (100)	0.1511
Female (%)	46 (10)	46 (10)	0 (0)	
Insured				
No (%)	112 (24)	109 (24)	3 (13)	0.2094
Yes (%)	356 (76)	336 (76)	20 (87)	
Malaria				
No (%)	182 (88)	170 (90)	12 (67)	**0.0116**
Yes (%)	25 (12)	19 (10)	6 (33)	
Anemia				
No (%)	67 (35)	64 (35)	3 (25)	0.5492
Yes (%)	127(65)	118 (65)	9 (75)	
Leukopenia				
No (%)	181 (93)	169 (93)	12 (100)	1.0000
Yes (%)	13 (7)	13 (7)	0 (0)	
Leukocytosis				
No (%)	102 (53)	95 (52)	7 (58)	0.6801
Yes (%)	92 (47)	87 (48)	5 (42)	
Congenital vs Acquired				
Acquired (%)	420 (90)	400 (90)	20 (87)	0.7202
Congenital (%)	48 (10)	45 (10)	3 (13)	
Procedure Category				
Gastrointestinal (%)	274 (59)	259 (58)	15 (65)	0.7268
Genitourinary (%)	61 (13)	59 (13)	2 (9)	
Injury (%)	71 (15)	69 (16)	2 (9)	
Miscellaneous (%)	32 (7)	30 (7)	2 (9)	
Neoplasm (%)	19 (4)	18 (4)	1 (4)	
Surgical Infection (%)	11 (2)	10 (2)	1 (4)	

Summarized in Table 8 are the multivariable logistic regression results for each of the outcomes variables. Gastrointestinal surgery, surgical traumas, surgical infections and lack of insurance were found to be independent predictors of prolonged length of stay ≥ 4 days. Male gender and older age were found to be inversely associated with in-hospital mortality. Finally, concurrent malaria infection was found to be an independent predictor of readmission within 90 days.

**Table 8:** Multivariable Analysis for the Predictors of Prolonged Length of Stay ≥ 4 Days, In-Hospital Mortality, and Readmission Within 90 Days

Independent Predictor of Outcomes	OR (95% CI)	*p*-Value	Area Under the Curve
**Prolonged Length of Stay ≥ 4 Days**			80%
Gastrointestinal	3.72 (1.92, 7.82)	0.0002	
Injury	3.13 (1.43, 7.27)	0.005	
Surgical Infection	12.12 (3.00, 62.64)	0.0009	
Insurance	0.41 (0.18, 0.94)	0.0351	
**In-Hospital Mortality**			98%
Male Gender	0.066 (0.005, 0.79)	0.0321	
Older Age	0.65 (0.50, 0.85)	0.0016	
**90-Day Readmission**			74%
Malaria	4.04 (1.28, 12.74)	0.0172	

## Discussion

This is the first study of perioperative outcomes in pediatric general surgery patients in rural Ghana. This study had several significant findings. First, that preoperative malaria infection is an actionable independent predictor of readmission in the pediatric surgical population in rural Ghana. Previous studies have shown that malaria is a leading cause of perioperative hyperpyrexia, increases the rate of surgical site infections, increases postoperative recovery and that surgery can cause reactivation malaria [23–28]. The burden of malaria infection in this region is highest in the pediatric population due to the immunological and behavioral drivers [29]. The underlying mechanism of readmission among patients with malaria is not fully understood. However, the pathophysiological changes caused by malaria infections, including excessive releases of cytokines and chemokines such as tumor necrosis factors and interleukin-2, could alter the healing process postoperatively [30]. Previous studies have suggested that surgery may weaken patients’ immune systems, increasing their susceptibility to malaria and its complications or to other pathogens [16].

Treating malaria preoperatively can lead to potential cost savings and reductions in morbidity. At ERH, the average cost is $16 USD per day of admission and an average length of stay of 4.3 days, the total direct cost of a hospital admission is approximately $69 USD not including transportation or lost wages. According to 2016 World Bank calculations, Ghana’s annual gross national income (GNI) per capita is $1840 USD with a Gini coefficient of 0.435 [31]. In 2013, 21.7 % of the population of the Eastern Region was living on less than 1,314 GHS ($241 USD) per adult per year [32]. Therefore, a $69 hospital admission would put a substantial financial burden on the family.

Consistent with previous studies, our study found an association between age and mortality [7, 11, 14, 19, 33, 34]. This may be the result of young children’s immature immune systems [35] or complex congenital disease processes [34]. It is likely that social processes common throughout sub-Saharan Africa such as lack of neonatal intensive care unit, lack of pediatric surgical specialists or delay in presentation contributed as well [19]. Due to the lack of pediatric surgical specialist capacity, all pediatric surgeries at ERH are performed by the general surgeons. Therefore, it is possible that infants and neonates with surgical conditions in this region are less likely to be brought to this hospital for treatment or are more likely to be transferred to the national hospital with pediatric surgery specialists, contributing to low numbers of infants and neonates operated on at this facility. In addition, we cannot rule out the possibility that having general surgeons performing the cases rather than pediatric surgical specialists may have contributed to the correlation between young age and mortality. To our surprise, we also found an association between female gender and perioperative mortality, which is in contrast with previous studies [5, 7, 19, 34, 36]. However, it is important to note that only 10 % of the patients in this study were female. Additional studies are needed to further investigate the relationship between gender and mortality in this region.

This study also identified several independent predictors of prolonged LOS, including gastrointestinal surgery, surgical injuries, surgical infections, and lack of insurance. It is to be expected that gastrointestinal surgery is associated with prolonged LOS, as many gastrointestinal procedures are major surgeries associated with an increased procedural complexity, increased risk of technical problems, longer surgical and anesthetic time, and immune deregulation resulting from the stress response to surgery or owing to impaired respiratory function in the postoperative period [37]. It is important to note that ERH does not have laparoscopy tools, so all procedures are performed using an open approach. In addition, surgical injuries can range from minor lacerations to complex polytraumas and surgical infections can range from localized abscesses to severe sepsis, with the more severe cases requiring extended admissions for medical management. However, it is possible that creating standardized management protocols can help to reduce the LOS in select cases. It is interesting to note that lack of insurance is associated with prolonged LOS. It is possible that lack of insurance may cause delayed presentation and therefore patients may present with more advanced disease, however it is also possible that patients without insurance are unable to pay their bills on time and therefore are kept in the hospital until their bills are paid. Additional studies need to be performed to further elucidate this association.

In comparing the results of this study to the literature, the average LOS for all pediatric surgical admissions in this study, 4.3 days, is similar to that reported by other studies [14, 38]. The overall in-hospital mortality rate of 1.7 % observed in this study was lower than the mortality rate of 12 % reported in a systematic review of pediatric surgeries in LMICs in Africa [6]. It is possible that this could be explained in part by region-specific differences, as the mortality rate in this study is similar to another West African study looking at pediatric general surgical cases in Nigeria, suggesting that there may be some genetic and environmental differences [19]. However, it is more likely that the pediatric surgical patients operated on at ERH represent a less acute and complex patient population as the most complex patients were likely referred to hospitals with pediatric surgery specialists.

This study had several limitations. First, this was a retrospective study and therefore is subject to a lack of sufficient clinical details and is potentially vulnerable to confounding. Second, ERH does not have a pediatric surgical specialist and therefore this study has an inherent selection bias, as very young or complex pediatric surgical patients were often referred to the national hospital for management by pediatric surgeons as evidenced by the low number of congenital cases operated on at this facility. This could have contributed to the low number of infants and neonates in our study, which in turn may have limited the power of our analysis. Third, there were only 8 documented mortalities in this study, which could limit the power of our analysis. The mortality data collected was limited to in-hospital mortality, and as a result, mortality rates could have been underestimated. Some patients could have died at home and would not have been captured in the hospital records. In addition, all patients who were admitted to the surgical ward but did not undergo a procedure were excluded, therefore if a patient died prior to their scheduled procedure, their death would not be reflected on the calculated in-hospital mortality rate. Finally, there were only two documented surgical site infections (SSI) in this study cohort, which was not enough to allow for meaningful analysis. It is possible that some cases of SSI were missed if patients failed to present for follow up. Consequently, it is recommended that a prospective multicenter study with detailed follow-up be performed to increase the precision and external validity of the results.

## Conclusion

In conclusion, this study revealed several areas for potential interventions to improve postoperative outcomes. First, malaria infection is a significant risk factor for readmission in the pediatric surgical population in rural Ghana. Preventing readmission in patients with malaria could lead to potential cost-savings and reductions in morbidity. Randomized control trials and prospective studies need to be completed to determine how to prevent readmission in patients with malaria. Improvements in the healthcare system to provide insurance to all patients and address other barriers to care may result in shorter hospital stays. Resources to provide laparoscopic equipment and training may reduce the length of stay for major gastrointestinal procedures. Improvements in the trauma system, including additional resources, additional training for staff, and a standardized multidisciplinary approach may result in improved management of injuries and reductions in the associated length of stay. Investment in workforce capacity building to train rural general surgeons, anesthetists and nurses to better address the specific perioperative needs of infants and young children, and ultimately training more pediatric surgeons to practice in regional and district hospitals, may result in lower pediatric surgery mortality rates in these settings. Future studies should also focus on further clarifying the association between female gender and mortality and the association between lack of insurance and prolonged length of stay in Ghana as well as to determine whether these associations are present in other regions.

## Data Availability

The datasets generated and analyzed during the current study are not publicly available to protect the privacy of the study participants but are available from the corresponding author on reasonable request.

## Abbreviations

LMICs: Low- and middle- income countries
SSI: Surgical site infections
ASA: American Society of Anesthesiologists
ERH: Eastern Regional Hospital
WBC: White Blood Cell count
LOS: Length of stay
HIV: Human immunodeficiency virus
Hgb: Hemoglobin
SD: Standard deviation
CI: Confidence interval
OR: Odds ratio
AUC: Area under the operating characteristic curve
IQR: Interquartile range
MO: Month
YRS: Years
VS: Versus
USD: US dollars
GNI: Gross national income
GHS: Ghana cedi (currency)
